# Experimental field evidence that out-group threats influence within-group behavior

**DOI:** 10.1093/beheco/arz095

**Published:** 2019-06-20

**Authors:** Amy Morris-Drake, Charlotte Christensen, Julie M Kern, Andrew N Radford

**Affiliations:** School of Biological Sciences, University of Bristol, Bristol, UK

**Keywords:** behavioral consequences, conflict, group living, out-group threat, rival group, within-group behavior

## Abstract

In social species, conspecific outsiders present various threats to groups and their members. These out-group threats are predicted to affect subsequent within-group interactions (e.g., affiliation and aggression) and individual behavior (e.g., foraging and vigilance decisions). However, experimental investigations of such consequences are rare, especially in natural conditions. We used field-based call playbacks and fecal presentations on habituated wild dwarf mongooses (*Helogale parvula*)—a cooperatively breeding, territorial species—to examine postinteraction responses to the simulated threat of a rival group. Dwarf mongooses invested more in grooming of groupmates, foraged closer together, and more regularly acted as sentinels (a raised guard) after encountering indicators of rival-group presence compared to control conditions. These behavioral changes likely arise from greater anxiety and, in the case of increased vigilance, the need to seek additional information about the threat. The influence of an out-group threat lasted at least 1 h but individuals of different dominance status and sex responded similarly, potentially because all group members suffer costs if a contest with rivals is lost. Our results provide field-based experimental evidence from wild animals that out-group threats can influence within-group behavior and decision making, and suggest the need for greater consideration of the lasting impacts of social conflict.

## Teaser

Threats from rival groups can lead to changes in within-group behavior. Using call playbacks and fecal presentations to simulate the presence of rivals, we show that wild dwarf mongooses groom groupmates for longer, forage closer together, and more regularly act as sentinels (raised guards) after encountering indicators of an out-group threat. These behavioral changes likely arise from greater anxiety and, in the case of sentinel behavior, the need to seek additional information about the threat.

## INTRODUCTION

In many social species, groups and their members face a variety of threats from conspecific outsiders but relatively little is known about the consequences of these so-called out-group threats. From hymenopterans to humans, individuals form stable permanent groups which often defend collective resources (Radford 2003; Kitchen and Beehner 2007; Batchelor and Briffa 2011; Christensen and Radford 2018). Out-group threats range from individuals seeking reproductive opportunities (Mares et al. 2011; Bruintjes et al. 2016), to single-sex groups looking to usurp dominant individuals (Ridley 2012), to whole groups attempting to acquire access to limited resources, such as food, mates, and sleeping sites (Wilson and Wrangham 2003; Kitchen and Beehner 2007; Golabek et al. 2012). A large literature exists on the immediate defensive behaviors and decisions relating to contests between rivals, considering who participates, the type of interaction (signaling exchanges to physical fighting), and what factors influence the outcome (Radford 2003; Radford and Du Plessis 2004; Majolo et al. 2005; Kitchen and Beehner 2007; Desjardins et al. 2008; Willems et al. 2015; Mirville et al. 2018). Far less attention has been paid to the wider consequences of out-group threats, beyond the actual interactions with outsiders or indicators of their presence (e.g., scent-marks), despite their importance for a full understanding of the costs and benefits of social conflict (Radford et al. 2016).

Alterations in within-group behavior in response to out-group threats are predicted, but experimental testing of these ideas is rare in nonhuman animals. There are strong theoretical arguments for why within-group affiliative and aggressive interactions are expected to change as a consequence of conflict with outsiders (Hamilton 1975; Reeve and Hölldobler 2007; Radford et al. 2016). From a proximate perspective, behavioral changes may result from conflict-induced increases in anxiety; functionally, affiliation may be used as a reward and to strengthen social bonds, while aggression may be a form of punishment (Radford et al. 2016). Correlational data have indicated an influence of out-group conflict: allopreening between green woodhoopoe (*Phoeniculus purpureus*) groupmates was elevated both immediately after intergroup contests and many hours later (Radford 2008a; Radford and Fawcett 2014), while vervet monkeys (*Chlorocebus pygerythrus*) exhibited greater levels of both within-group affiliation and aggression during extended bouts of intergroup conflict (Arseneau-Robar et al. 2016, 2018). In captive experiments, cichlid fish (*Neolamprologus pulcher*) increased affiliative interactions with groupmates after simulated intrusions by out-group rivals (Bruintjes et al. 2016), and tufted capuchin monkeys (*Cebus apella)* increased aggression levels during, but not after, out-group encounters (Polizzi di Sorrentino et al. 2012). However, we know of only one experimental study testing these ideas in the wild: green woodhoopoes increased their allopreening more following playback of vocal choruses from non-neighboring groups compared with those from neighboring groups (Radford 2008b). Moreover, changes in behaviors other than affiliation and aggression are expected in response to out-group conflict (Radford et al. 2016). While research on individual and pair-bonded territory holders has shown, for instance, increased vigilance and reduced foraging following territorial intrusions (Olendorf et al. 2004; Descovich et al. 2012), experimental tests of such effects have not been conducted in group-living species (Christensen and Radford 2018).

Postinteraction responses to out-group threats are expected to differ depending on the characteristics of individual group members. The interests and motivations of group members are unlikely to be perfectly aligned because of differences in, for example, sex, age, kinship, and dominance status (Heinsohn and Packer 1995; Radford et al. 2016). It is well known that individuals vary in their levels of defensive participation when encountering an out-group threat (Majolo et al. 2005; Kitchen and Beehner 2007; Schindler and Radford 2018). However, few studies have empirically tested how groupmates differ in their postinteraction behavior (Radford et al. 2016). There are 2 examples where the effect of dominance status has been explored: correlational data from wild green woodhoopoes indicated that the postinteraction increase in affiliation is driven by the dominant breeding pair (Radford 2008a), whereas the equivalent affiliation increase seen in captive cichlids is driven by subordinates (Bruintjes et al. 2016). Observational data from wild vervet monkeys has been used to consider sex differences in within-group affiliation and aggression during intergroup encounters (Arseneau-Robar et al. 2016, 2018). Field experiments are now needed to investigate further how within-group dynamics are affected by out-group threats.

The level of perceived out-group threat is also likely to affect postinteraction behavior. One well-studied contributor to threat level is rival-group identity: in some species, strangers are a greater threat than neighbors (resulting in a dear–enemy effect); in other cases, neighbors are more of a threat than strangers (the nasty-neighbor effect) (Radford 2005; Müller and Manser 2007; Christensen and Radford 2018). These differences have been shown to influence postinteraction behavior in green woodhoopoes and cichlid fish (Radford 2008b; Bruintjes et al. 2016). Another element of threat level is the intensity of an intergroup interaction, which can range from the exchanging of information (Mirville et al. 2018) through signaling contests (Radford 2003) to physical fights (Mitani et al. 2010). There is some evidence from correlational data that interaction intensity can affect subsequent behavior (Radford 2008a; Radford and Fawcett 2014). A less-considered aspect of threat level is the likelihood of an out-group contest arising (Radford 2011). Various cues can provide information on the current or recent presence of rivals. For instance, many social species produce regular within-group vocalizations (Palombit et al. 1999; Radford and Ridley 2008; Townsend et al. 2011) which would reveal the proximity of a rival group; mammals commonly demarcate their territorial boundaries by depositing scent-marks (e.g., urine, feces, anal gland secretions) at communal latrines (Brown and Macdonald 1985), indicating rival presence sometime in the past. Animals might, therefore, be expected to behave differently following detection of cues that indicate different likelihoods of an imminent out-group contest.

In this study, we experimentally investigate within-group behavioral responses to out-group threats in wild dwarf mongooses (*Helogale parvula*), an ideal model species for biological and logistical reasons. Dwarf mongooses are cooperative breeders, living in family groups comprising a dominant breeding pair and nonbreeding subordinates of both sexes (Rasa 1977). Group members sleep, forage, and travel together within a shared territory (Rood 1983; Kern and Radford 2013; Christensen et al. 2016). At the sleeping burrow in the morning and evening, within-group affiliation is displayed frequently via the grooming of others (Kern and Radford 2016, 2018). Throughout the day, individuals make constant decisions relating to foraging (e.g., how close to forage near a groupmate; Kern JM, Radford AN, unpublished data) and vigilance (e.g., whether and when to act as a sentinel; Kern and Radford 2014, 2017). Each group has one or more conspecific neighbors; territorial behavior involves scent marking at communal latrines and physical defense when rivals are encountered (Rasa 1973; Christensen et al. 2016). Latrines are usually visited as a group and scent-marks (urine, feces, cheek gland, and anal gland secretions) are deposited by multiple group members. The ability to habituate wild dwarf mongooses to the close presence of human observers allows the collection of ecologically valid data and the running of experiments in natural conditions (Kern and Radford 2013, 2014, 2016, 2018; Christensen et al. 2016; Morris-Drake et al. 2016, 2017).

We conducted 2 field-based experimental manipulations to determine individual behavioral responses to out-group threats. First, we considered how affiliative (grooming) and aggressive within-group interactions are affected by a simulated out-group threat (playback of close calls to indicate a rival group nearby). Second, we considered whether the threat of a rival group influences individual foraging and vigilance decisions, and how those responses are affected by potential variation in the threat level; we played back rival-group close calls to represent a threat from a nearby group and presented rival-group feces to represent a lesser threat (as those who deposited the feces may have moved away). We predicted that, as a consequence of increased anxiety and need for additional information about the threat, individuals would display more within-group grooming and aggression, forage closer together and contribute more to sentinel behavior after the simulated presence of a rival group compared to control conditions. We expected dominant individuals to show stronger responses than subordinates as the former are likely to suffer the greatest costs if rival groups gain access to limited resources (e.g., food, mates, and sleeping sites). We also expected females to contribute more than males after rival treatments because females are the philopatric sex in dwarf mongooses (Rood 1987); the retention or loss of resources has potentially longer-term consequences for the philopatric sex. Finally, we predicted a stronger response to rival-group call playbacks than fecal presentations as the former could indicate the possibility of a more imminent contest.

## METHODS

### Study site and population

Data were collected from a habituated wild population of dwarf mongooses as part of the long-term Dwarf Mongoose Research Project (DMRP). This work was conducted on Sorabi Rock Lodge, a private game reserve in the Limpopo Province, South Africa (24° 11′S, 30° 46′E); full details available in Kern and Radford (2013). Experimental data were collected over 2 periods (October 2015 to February 2016 and July–September 2017) on 7 wild groups (mean ± SD group size: 10.9 ± 5.2, range: 4–17); data were obtained from all habituated groups available at the time. Groups were habituated to close human presence (<5 m), facilitating controlled experimental manipulations in natural conditions. All work was conducted under permission from the Department of Environmental Affairs and Tourism, Limpopo Province (permit number: 001-CPM403-00013) and the Ethical Review Group, University of Bristol (University Investigator Number: UIN/17/074).

Individuals can be identified from distinctive physical features or from small marks of blonde dye (Wella UK Ltd, Surrey, UK) applied to their fur (Kern and Radford 2013). The population has been studied since 2011 and the dominance status and sex of all individuals is therefore known. The dominant pair in a group are recognized through observations of aggressive behavior, foraging displacements, and scent marking, while individuals are sexed through observations of ano-genital grooming (Kern and Radford 2016). Data were only collected from adults (individuals older than 12 months) as juveniles do not routinely engage in at least some of the measured behaviors (e.g., sentinel activity) (Kern et al. 2016).

### Field experiments

Two field-based experiments were conducted to investigate within-group behavioral responses to simulated out-group threats. In Experiment 1, 7 groups each received 2 treatments at their morning sleeping burrow: 1) playback of the close calls of a non-neighboring group (one that did not share any territorial boundaries with the focal group) and 2) playback of herbivore grunts and huffs (as a control). The immediate responses to the playback and subsequent within-group affiliative (grooming) and aggressive interactions were recorded. In Experiment 2, 7 groups each received 4 treatments while foraging: 1) playback of the close calls of a non-neighboring group; 2) playback of herbivore grunts and huffs (as a control); 3) presentation of feces from the same non-neighboring group as in (1); and 4) presentation of herbivore feces (as a control). The immediate responses to playback and fecal presentations, as well as subsequent foraging and vigilance decisions, were recorded.

### Playback and fecal stimuli

Playback stimuli were constructed from original sound recordings. All sound recordings were made with a Marantz PMD660 professional solid-state recorder (Marantz America, Mahwah, NJ) and a Sennheiser directional microphone (Sennheiser UK, High Wycombe, Buckinghamshire, UK) with a Rycote softie windshield (Rycote Microphone Windshields, Stroud, Gloucestershire, UK). Recordings were made at a sampling rate of 48 kHz with a 24-bit resolution and stored on a Transcend SD card (Transcend, Taipei, Taiwan). Dwarf mongooses are very vocal and rely on acoustic communication to coordinate their cooperative behaviors; they produce close calls (low-amplitude vocalizations) continuously while foraging and moving (Sharpe et al. 2013). Close calls therefore provide a vocal cue as to the presence of another group; unlike some other species (Radford 2003; Golabek and Radford 2013), dwarf mongooses do not produce a particular vocalization during encounters with rival groups that indicates more directly an out-group threat (Amy Morris-Drake personal observation). Close calls were recorded ad libitum from 4 randomly chosen adult individuals in each group, including one or both dominants and either 2 or 3 subordinates accordingly. Recordings were made from 1 to 2 m during behavioral observation sessions in calm weather conditions. The peak sound-pressure level (SPLA) of close calls was measured (in dB) using a HandyMAN TEK 1345 sound meter (Metrel UK Ltd., Normanton, UK) to standardize playback volume at natural levels in experimental trials. Herbivore sounds were recorded in calm weather conditions from the vicinity of the main lodge at the study site, where a variety of ungulate species, including zebra (*Equus quagga*), giraffe (*Giraffa camelopardalis giraffe*), blue wildebeest (*Connochaetes taurinus*), and waterbuck (*Kobus ellipsiprymnus*), are accustomed to human presence. The microphone was attached to a tree 10 m from an artificial feeding area and left to record for 1 h.

Five-minute playback tracks were constructed in Audacity (version 2.1.3). For rival-group tracks, close calls with good signal-to-noise ratio were randomly chosen and extracted from original recordings. Four different call sequences were constructed per group, with each sequence consisting of one close call from each of the 4 recorded individuals. These sequences were selected in a random order and inserted into a 12-s block of ambient sound; ambient-sound recordings were made from the center of the relevant territory with the equipment described above. Five such 12 s blocks were edited together, and this 1 min block was copied 5 times to create a 5-min track. Rival-group tracks had a close-call rate of 75 calls per minute, which is the natural vocalization rate of 4 dwarf mongooses (Sharpe et al. 2013). Control tracks consisted of randomly chosen herbivore sounds (zebra and wildebeest grunts or huffs) with good signal-to-noise ratio that were extracted from original recordings. For each track, 4 different sequences were generated, each consisting of 4 unique herbivore sounds. These sequences were randomly selected and inserted into five 12 s blocks of ambient sound, which were then copied to create a 5 min track with 20 herbivore sounds per minute. In all playback tracks, sounds were gradually faded in with increasing amplitude to simulate an approach. At the midway point of each track, the amplitude was 55 dB SPLA at 1 m, which is the natural volume of dwarf mongoose close calls (see Playback and fecal stimuli). Different rival-group and control tracks were constructed for trials to different groups.

Fecal collection, storage, and usage followed the protocol previously used on this study population by Christensen et al. (2016). Freshly deposited dwarf mongoose feces were collected immediately and placed in airtight plastic bags inside glass pots while in the field. Feces were refrigerated (5 °C) on return to the field base and always used in an experimental presentation the following day. Each presented sample consisted of 1 deposit from 4 different adult individuals, including at least 1 dominant group member. For the control treatment, 4 fresh waterbuck or giraffe fecal pellets (both similar in diameter to dwarf mongoose feces) were collected from the vicinity of the main lodge at the study site. Storage and usage protocols matched those for dwarf mongoose feces. Different rival-group and control feces were used for each trial.

### General experimental protocol

For each experiment, trials to a given group were carried out on separate days and completed within 1 week for Experiment 1 (mean ± SE = 3.4 ± 0.7 days, range = 1–5 days) and 1 month for Experiment 2 (mean ± SE = 11.8 ± 3.3 days, range = 4–30 days). Treatment order was counterbalanced between groups. Trials were not conducted if there had been an intergroup interaction earlier that day and were abandoned if an alarm call or any other group disturbance (e.g., snake mob) occurred during the experimental manipulation (Experiment 1: *N* = 3; Experiment 2: *N* = 5). Abandoned trials were rerun another day when the above conditions were met. Behavioral responses in all trials were recorded to a Dictaphone (ICD-PX312, Sony, Sony Europe Limited, Surrey, UK); collecting data through live observation allowed a wider field of view and consideration of more group members than video recording, but precluded blind scoring. The location of each experimental manipulation was recorded using a GPS (Garmin Etrex H GPS; Garmin Europe Ltd, Southampton, Hampshire, UK). Analysis of the GPS data revealed that there was no significant difference between the different treatments in an experiment in the likelihood that they were run in the core or the periphery of the focal group’s territory (see Supplementary Material).

Playback trials followed our standard general protocol (Kern and Radford 2014, 2017, 2018). Trials took place when there had been no alarm call or group disturbance for at least 10 min. Tracks were played from an iPod (Apple, Cupertino, CA) through a portable SME-AFS field loudspeaker (Saul Mineroff Electronics Inc., New York, NY), which was concealed in vegetation (Experiment 1: near the sleeping burrow; Experiment 2: near the foraging group). The mongooses were attracted to a location 5 m from the loudspeaker using a small amount of hard-boiled egg. Once 50% of the adults in the group were present, the relevant playback track was started and dictation of behavior commenced. The following immediate responses to the playback were determined for adults within 5 m of the loudspeaker: whether an individual looked and orientated (whole body pointing towards the loudspeaker) in the direction of the loudspeaker; and whether an individual interrupted foraging and directly approached the loudspeaker.

Fecal trials followed the general protocol in Christensen et al. (2016), with presentations conducted at known dwarf mongoose latrines. Once the group left the morning sleeping burrow to start foraging, the presence of nearby latrines (recorded as part of DMRP daily data collection) was tracked using the map page on the GPS. When the group appeared to be approaching a known latrine, the observer moved ahead and placed the relevant fecal samples at the site. The observer then attracted the mongooses to a location 5 m from the latrine using a small amount of hard-boiled egg. Once 50% of the adults in the group were present, dictation of behavior commenced; a trial was deemed to have started once the first individual approached the latrine. The following immediate responses of adults to the fecal presentation were determined: the number and identity of individuals that participated in the latrine; and the number and duration of all occasions that individuals sniffed the presentation.

### Specific protocols for individual experiments

For Experiment 1, both sound treatments to the same group (rival-group playback and control playback) were conducted at the same type of sleeping burrow (always termite mounds) when weather conditions were calm. The playback equipment was set up before the first mongoose emerged; the field loudspeaker was hidden from view 5 m from the burrow. Two minutes after all the group members had emerged, the mongooses were attracted to a location 5 m from the loudspeaker and the trial commenced (see *General experimental protocol*). When the playback track ended, data on within-group behavioral interactions were dictated until 50% of the group had left to start foraging. All aggressive and affiliative (grooming) interactions between adult individuals were recorded, including the identity of those involved and the duration of the interactive bout.

For Experiment 2, all 4 treatments to the same group (rival-group and control playbacks and presentations of rival-group and control feces) were conducted when the group was foraging in a similar habitat type during calm weather conditions. Trials were run as per the *General experimental protocol*. Groups were followed for an hour after the experimental manipulations, during which nearest-neighbor scans were conducted every 10 min and sentinel scans were conducted every 5 min. Nearest-neighbor scans entailed estimating the distance (to the nearest 0.5 m) of the closest group member to all foraging individuals in sight; it was not possible to record individual identities regularly without disrupting foragers. Sentinel scans entailed noting whether a sentinel was present and, if so, its identity and whether it was facing in the direction of the experimental manipulation.

### Data analysis

Mixed models were constructed in RStudio 3.2.2 (R Development Core Team 2012), while all other analyses were run using IBM SPSS Statistics for Windows, version 24 (IBM Corp, 2016). All tests were 2-tailed and considered significant at *P* < 0.05. Parametric tests were used where the residuals fitted the relevant assumptions of normality and homogeneity of variance. Logarithmic and arcsine transformations were conducted to achieve normality of errors in some cases (details below); otherwise nonparametric tests were used.

For simple paired data, we used Wilcoxon signed-rank tests. As sample sizes were small, *P*-value calculations based on the default asymptotic distribution of the test statistic would be unreliable; we therefore used the Monte Carlo resampling method (based on 10,000 samples) to generate *P*-values. In cases where multiple factors needed to be taken into consideration, repeated-measures analysis of variances (ANOVAs), linear mixed models (LMMs), or generalized linear mixed models (GLMMs) (package: lme4; Bates et al. 2015) were used. Mixed models contain fixed and random effects, the latter accounting for repeated trials to the same group or individual within a group. When running mixed models, all explanatory terms and 2-way interactions of interest were included in the maximal model. Models were refined using Akaike Information Criterion comparisons between candidate model structures, combined with stepwise deletion of nonsignificant terms (Crawley 2007). The minimal model only contained terms that explained significant variation in the data. *P*-values were estimated using the drop1 command (using the lmerTest package version 3.1–0 for LMMs) and a graphical approach was used to confirm normality and homoscedasticity of residuals.

### Experiment 1

To determine if rival-group playbacks induced an increased response relative to control playbacks, and thus simulate an out-group threat as planned, the immediate responses were considered. Sound-treatment differences in the proportion of individuals that looked and orientated towards the loudspeaker and the proportion of individuals that directly approached the loudspeaker were analyzed using Wilcoxon signed-ranks tests.

To examine the influence of sound treatment on subsequent within-group affiliative interactions (there were no aggressive interactions observed), grooming bouts of >5 s were analyzed in 2 stages. First, differences in the overall rate (total number of grooming bouts divided by duration at sleeping burrow) and mean duration of grooming bouts (using all data from each trial combined) were considered using Wilcoxon signed-ranks tests. Second, whether the significant difference in grooming bout duration (see Results for details) was driven by individuals of different dominance status or sex was considered. Two LMMs (1 for dominance status and 1 for sex), with identity link functions, were run on the raw data. These included sound treatment (rival group, control) and either dominance status (dominant, subordinate) or sex (female, male), as well as their interaction with sound treatment, as fixed effects; individual identity was nested within group identity as the random term.

### Experiment 2

To determine if rival-group playbacks and fecal presentations induced an increased response relative to control playbacks and fecal presentations, and thus simulate an out-group threat as planned, the immediate responses were considered. For the playback trials, sound-treatment differences in the proportion of individuals that looked towards the loudspeaker and the proportion of individuals that directly approached the loudspeaker were analyzed using Wilcoxon signed-ranks tests. For the fecal trials, presentation-treatment differences in the proportion of the group that participated in the latrine and the total time spent sniffing the feces were analyzed using Wilcoxon signed-ranks tests.

To examine the influence of experimental treatment on subsequent within-group foraging decisions, log-transformed mean nearest-neighbor distances during the postmanipulation hour were analyzed in a 2×2 repeated-measures ANOVA. Intruder identity (rival group, control), manipulation type (playback, fecal presentation), and their interaction were included as predictor variables. To determine if the experimental treatment had a lasting effect, the nearest-neighbor foraging distances from the first and last scans (10 and 60 min postmanipulation, respectively) were compared using a second 2×2 repeated-measures ANOVA. The first analysis revealed no difference in nearest-neighbor distances depending on manipulation type (see Results for details); so, means were calculated from the 2 rival-group treatments and the 2 control treatments for the second ANOVA. Intruder identity (rival group, control), scan period (10 min postmanipulation, 60 min postmanipulation), and their interaction were included as predictor variables in this second ANOVA.

To examine the influence of experimental treatment on subsequent within-group sentinel decisions, arc-sine-square-root-transformed proportions of scan samples in which a sentinel was present were analyzed in a 2×2 repeated-measures ANOVA. Intruder identity (rival group, control), manipulation type (playback, fecal presentation), and their interaction were included as predictor variables. The likelihood of sentinels facing in the direction of the experimental manipulation was analyzed using a Wilcoxon signed-ranks test for just the period after call playbacks; no individuals acted as a sentinel in the period after a control fecal presentation. Whether the significant difference in sentinel occurrence (see Results for details) was driven by individuals of different dominance status or sex was then considered. Two GLMMs (1 for dominance status and 1 for sex), with binomial error distributions and logit-link functions, were run. Since the first analysis of sentinel behavior revealed no difference depending on manipulation type (see Results for details), values were combined for the 2 rival-group treatments and the 2 control treatments. The models bound the number of scan samples in which an individual was on sentinel duty with the number of scan samples in which the individual was not acting as a sentinel, testing the likelihood of an individual being on sentinel duty over a given period. The fixed effects applied to these models were intruder identity (rival group, control) and either dominance status (dominant, subordinate) or sex (female, male), as well as their interaction with intruder identity; individual identity was nested within group identity as the random term.

## RESULTS

### Experiment 1

**Figure 1:**
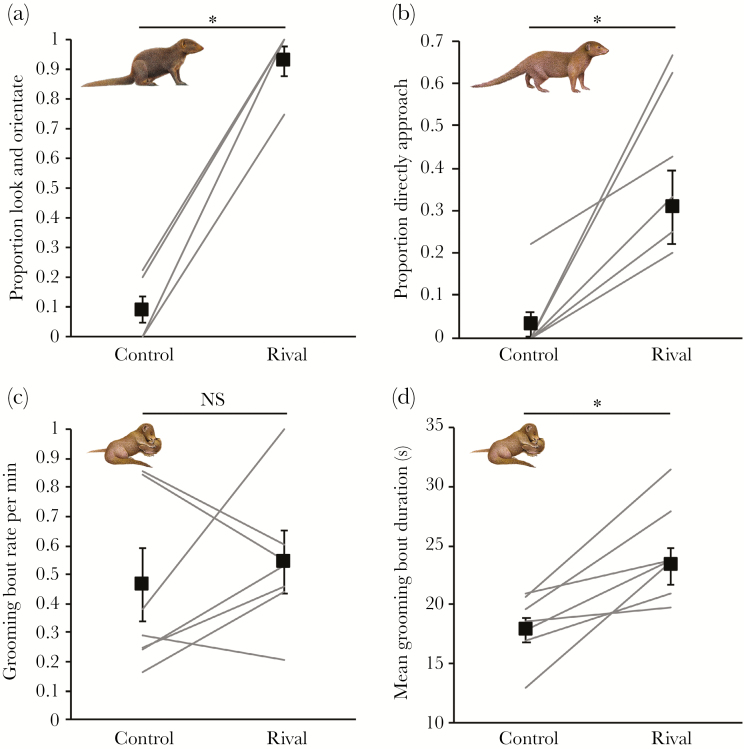
Immediate (A, B) and subsequent (C, D) responses of dwarf mongooses to control (herbivore) and rival-group playbacks (*N* = 7 groups). Shown in all panels are the values for each group (connected by solid lines; the data values for some groups are the same, thus the number of lines can appear less than 7) and the overall treatment mean (solid squares) ± SE. NS = nonsignificant. *P < 0.05.

The immediate responses to playback at the sleeping burrow were significantly affected by sound treatment. A greater proportion of individuals looked and orientated towards the loudspeaker during rival-group playback compared with control playback (Wilcoxon signed-ranks test: Z = 2.379, *N* = 7, Monte Carlo *P* = 0.014; Figure 1A). A greater proportion of individuals also approached the loudspeaker directly during playback of a rival group compared with control playback (Z = 2.201, *N* = 7, Monte Carlo *P* = 0.030; Figure 1B).

Subsequent within-group responses to playback were significantly affected by sound treatment. No aggressive interactions were observed following either sound treatment, but affiliative (grooming) interactions were common (mean ± SD grooming rate: 0.5 ± 0.3 bouts per minute). Although there was no significant sound-treatment difference in the overall rate of grooming interactions (Wilcoxon signed-ranks test: Z = 0.676, *N* = 7, Monte Carlo *P* = 0.576; Figure 1C), grooming bouts were longer after playback of a rival group compared with a control playback (Z = 2.366, *N* = 7, Monte Carlo *P* = 0.015; Figure 1D). Grooming-bout duration was not significantly affected by the interaction between treatment type (control, rival) and either dominance status (LMM: χ^2^ = 0.348, df = 1, *P* = 0.560; Table 1) or sex (χ^2^ = 0.001, df = 1, *P* = 0.973; Table 1); dominant and subordinate individuals responded similarly to the out-group threat, as did males and females.

**Table 1:** Output from LMMs (A,B) and GLMMs (C,D) investigating whether the significant difference in grooming bout duration was driven by individuals of different dominance status or sex (A,B) and whether the significant difference in sentinel occurrence was driven by individuals of different dominance status or sex (C,D)

	Fixed effect	Estimate ± SE	df	χ^2^	*P*
**(A) Mean grooming bout duration by dominance status**					
Random terms	*Group ID*	1.328 ± 1.153			
	*Individual ID in Group*	6.113 ± 2.472			
Minimal model	(Intercept)	18.423 ± 1.520			
	**Trial**	**7.164 ± 1.949**	**1**	**13.514**	**<0.001**
Dropped terms	Trial:Status		1	0.348	0.560
	Status		1	0.022	0.884
**(B) Mean grooming bout duration by sex**					
Random terms	*Group ID*	1.328 ± 1.153			
	*Individual ID in Group*	6.113 ± 2.472			
Minimal model	(Intercept)	18.423 ± 1.520			
	**Trial**	**7.164 ± 1.949**	**1**	**13.514**	**<0.001**
Dropped terms	Trial:Sex		1	0.001	0.973
	Sex		1	0.134	0.718
**(C) Proportion of scan samples with a sentinel present by dominance status**					
Random terms	*Group ID*	0.421 ± 0.649			
	*Individual ID in Group*	0.002 ± 0.043			
Minimal model	(Intercept)	3.111 ± 0.284			
	**Trial**	**1.837 ± 0.211**	**1**	**100.462**	**<0.001**
	**Status**	**0.956 ± 0.287**	**1**	**9.717**	**0.002**
Dropped terms	Trial:Status		1	0.901	0.343
**(D) Proportion of scan samples with a sentinel present by sex**					
Random terms	*Group ID*	0.636 ± 0.797			
	*Individual ID in Group*	0.000 ± 0.000			
Minimal model	(Intercept)	3.769 ± 0.002			
	**Trial**	**1.836 ± 0.002**	**1**	**100.400**	**<0.001**
Dropped terms	Trial:Sex		1	1.815	0.178
	Sex		1	0.956	0.328

Significant fixed terms shown in bold; variance ± SD reported for random terms (in italics).

### Experiment 2

**Figure 2:**
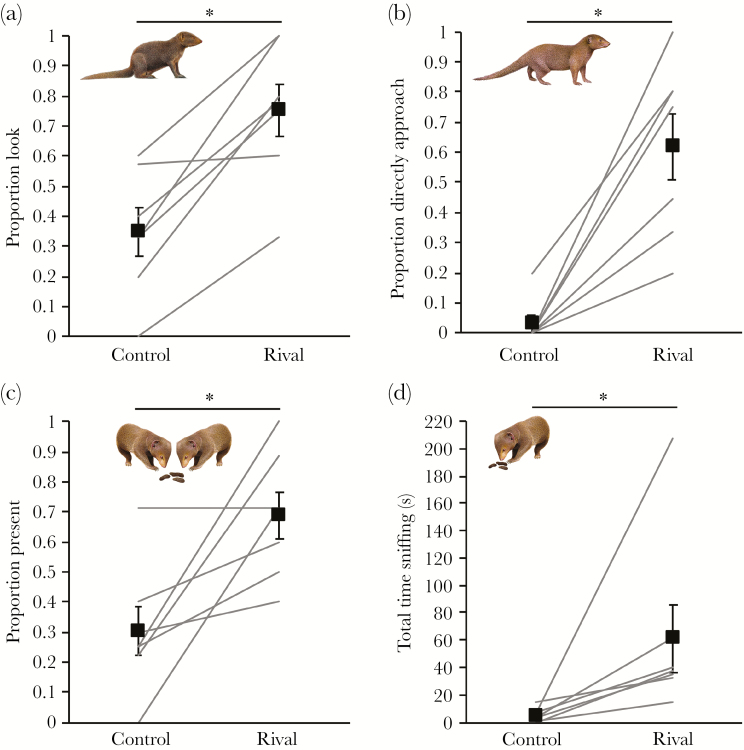
Immediate responses of dwarf mongooses to control (herbivore) and rival-group (A, B) playbacks and (C, D) fecal presentations (*N* = 7 groups). Shown in all panels are the values for each group (connected by solid lines) and the overall treatment mean (solid squares) ± SE. *P < 0.05.

The immediate responses to experimental trials were significantly affected by intruder identity. As in Experiment 1, a greater proportion of individuals looked at the loudspeaker (Wilcoxon signed-ranks test: Z = 2.366, *N* = 7, Monte Carlo *P* = 0.015; Figure 2A) and directly approached the loudspeaker (Z = 2.366, *N* = 7, Monte Carlo *P* = 0.015; Figure 2B) during rival-group playback compared with control playback. As in Christensen et al. (2016), there was a significantly greater proportion of individuals participating in the latrine event (Z = 2.201, *N* = 7, Monte Carlo *P* = 0.035; Figure 2C) and a significantly longer total time spent sniffing the feces (Z = 2.366, *N* = 7, Monte Carlo *P* = 0.015; Figure 2D) in response to rival-group feces compared with control feces.

**Figure 3:**
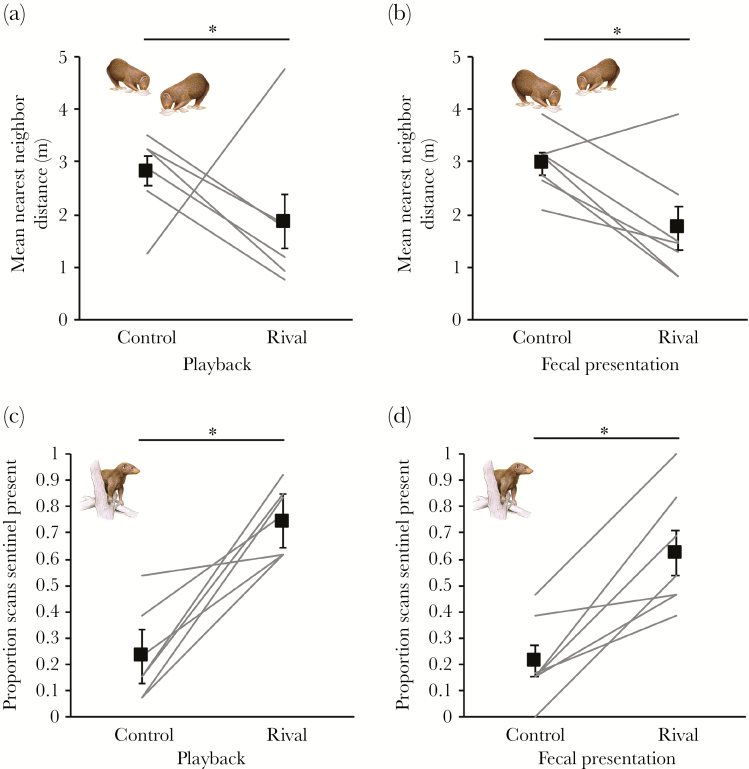
Foraging (A, B) and sentinel (C, D) responses of dwarf mongooses to control (herbivore) and rival-group playbacks and fecal presentations (*N* = 7 groups). Shown in all panels are the values for each group (connected by solid lines) and the overall treatment mean (solid squares) ± SE. *P < 0.05.

Foraging decisions in the hour after the manipulation were affected by intruder identity (rival, control), but not manipulation type (playback, fecal presentation). Overall, individuals foraged significantly closer to another group member following rival-group playbacks and fecal presentations compared to control treatments (repeated-measures ANOVA: *F*_1,6_ = 8.995, *P* = 0.024; Figure 3A,B), irrespective of manipulation type (main effect: *F*_1,6_ = 0.017, *P* = 0.900; interaction with intruder identity: *F*_1,6_ = 0.107, *P* = 0.755). The stronger response to rival-group treatments compared with control treatments lasted for at least 1 h after the simulated intrusion: there was no significant effect of scan period (10 min, 60 min postmanipulation) on nearest-neighbor distances (*F*_1,6_ = 0.046, *P* = 0.838; interaction with intruder identity: *F*_1,6_ = 0.677, *P* = 0.442).

Sentinel decisions in the hour after the manipulation were similarly affected by intruder identity. Overall, there was significantly more sentinel behavior following rival-group playbacks and fecal presentations compared to control treatments (repeated-measures ANOVA: *F*_1,6_ = 30.274, *P* = 0.002; Figure 3C,D), irrespective of manipulation type (main effect: *F*_1,6_ = 0.542, *P* = 0.489; interaction with intruder identity: *F*_1,6_ = 0.270, *P* = 0.622). Moreover, a greater proportion of sentinels were facing in the direction of the manipulation after rival-group playback compared with control playback (Wilcoxon signed-ranks test: Z = 2.366, *N* = 7, Monte Carlo *P* = 0.015). There was no significant effect of the interaction between treatment type (control, rival) and either dominance status (GLMM: χ^2^ = 0.901, df = 1, *P* = 0.343; Table 1) or sex (χ^2^ = 1.815, df = 1, *P* = 0.178; Table 1) on postmanipulation sentinel behavior; dominant and subordinate individuals responded similarly to the out-group threat, as did males and females.

## DISCUSSION

Following a simulated threat from a rival group, dwarf mongooses invested more in grooming, foraged closer together and conducted more sentinel behavior than in control trials. Previous observational studies of birds and primates (Radford 2008a; Arseneau-Robar et al. 2016), and an experimental study with captive fish (Bruintjes et al. 2016), have also found an increase in affiliation among groupmates as a consequence of out-group conflict; alterations in foraging and vigilance have not previously been examined in an out-group context. In principle, the behavioral changes following rival-group playbacks and fecal presentations cf. control treatments could simply be a response to any mongoose stimulus. However, Christensen et al. (2016) showed a stronger response to rival-group feces than own-group feces, with no difference in response to herbivore and own-group feces, and we found a similar pattern of responses to rival-group, own-group, and herbivore playbacks (Supplementary Material). We are therefore confident that our results represent a response to rival-group stimuli rather than mongoose stimuli per se, and that our study provides rare experimental evidence from a wild population that out-group threats influence within-group behavior (see also Radford 2008b).

Increased anxiety arising from out-group conflict is likely to play a role in driving changes to within-group interactions. Conflict induces anxiety, not least because of the risks of potential injury or death, disrupted relationships or lost resources (Aureli et al. 2002; Radford et al. 2016). A proximate reason for increased postinteraction affiliation (such as the elevated grooming observed in the mongooses) is anxiety reduction, since both the giving and receiving of grooming is known to have this benefit in mammals and birds (von Holst 1998; Aureli et al. 1999; Aureli and Yates 2010; Radford 2012). Functionally, increased affiliation could act as a reward for recent participation and/or as an incentive for future help in interactions with out-group rivals (Radford 2008a, 2011; Arseneau-Robar et al. 2016; Radford et al. 2016). While greater anxiety might potentially also lead to increased within-group aggression, either as a byproduct or if it is used to punish free-riders (Arseneau-Robar et al. 2016, 2018; Radford et al. 2016), no aggression was seen between dwarf mongoose groupmates following rival-group playbacks at the sleeping burrow. As within-group aggression is generally more prevalent in this species when group members are foraging (Amy Morris-Drake personal observation), perhaps an effect of out-group conflict on antagonistic interactions might be observed at these times.

Foraging closer to others in the aftermath of a simulated out-group threat may also be a consequence of increased anxiety. As is the case following within-group conflict (Verbeek and de Waal 1997; Mallavarapu et al. 2006), close proximity to other group members could function directly to reduce anxiety. Moreover, as in response to increased predation risk (e.g., Bell et al. 2009), groupmates may forage closer together when there is a higher likelihood of a contest with outsiders if that means enhanced support or some dilution of the personal risk. These benefits might be particularly apparent if individuals forage near close affiliates (Young et al. 2014); the existence of close affiliations can generally lower anxiety levels through social buffering (Cohen and Wills 1985; Wittig et al. 2008). Dwarf mongooses exhibit social bonds of different strengths with different groupmates (Kern and Radford 2016), but future work would be needed to examine such detailed foraging relationships following stressful events.

Increased vigilance following cues of rival group presence could result from greater anxiety or a need to gather more information about the threat. Sentinels are suggested to be in a safer position than foragers, at least from a predatory threat (Bednekoff 1997; Wright et al. 2001). Whether the same applies in an out-group context is unknown, but if this was driving the changes seen then several individuals might be expected to adopt a raised position at the same time and that was rarely the case in our study. More likely, perhaps, is that the increase in sentinel activity reflects an attempt to obtain additional information. Traditionally, sentinel behavior is discussed in an antipredator context (Bednekoff 2015). However, individuals may also act as sentinels for other reasons, such as to gain information about dispersal or mating opportunities (Walker et al. 2016). In the current context, they may do so because there has been an indication of a rival group (from secondary cues such as vocalizations or feces) but no visual sign of those outsiders. Our experimental manipulations represent a likely common occurrence as dwarf mongooses regularly encounter feces of other groups at latrines (Christensen et al. 2016) and the thick vegetation may mean that lines of sight are obscured and the producers of vocalizations cannot easily be detected visually, especially by foragers on the ground. Information on the location of the group that is calling or has deposited feces, as well as other knowledge such as their group size, is likely valuable in terms of subsequent decision making.

The few previous empirical studies on the consequences of out-group conflict have tended to focus on just whether there is an effect in the immediate aftermath of an interaction. As an exception, Radford and Fawcett (2014) provided correlational evidence that out-group contests affect decision making and group cohesion over the course of a day. Green woodhoopoe groups that had an intense intergroup interaction in the morning were more likely to roost in the zone of conflict that evening, in addition to being more likely to roost together and to preen one another. Here, we show experimentally that individuals are still foraging closer together (a response to encountering cues of rival-group presence) at least an hour after the manipulation. Investigations of longer-term responses, beyond the immediate effect of elevated anxiety, are crucial if we are to understand fully the range of costs and benefits at play and will help to shed light on the relationship between intergroup conflict and its suggested role in the evolution of cooperation.

All adults invested in more grooming and sentinel behavior after exposure to an out-group threat, regardless of their dominance status and sex. This contrasts previous observational and captive work on woodhoopoes and cichlid fish, which found differences in affiliation between individuals of different dominance status (Radford 2008a; Bruintjes et al. 2016). There are at least 2 possible explanations for a lack of such a finding in our mongoose work. In the cichlid study, there were actual intruders which elicited aggressive defensive actions; in the woodhoopoe study, the playback was of a chorus vocalization used in adversarial encounters. By contrast, our experiments provided cues to current or recent rival presence; they may not have elicited a full defensive response. Perhaps, some dominance or sex variation would be seen in dwarf mongooses if the out-group interactions escalated. A second potential explanation relates to the perceived threat. The intrusion of a rival group could prove costly for all groupmates if the former are seeking to annex shared resources, such as food, sleeping sites, or part of the territory (Wilson and Wrangham 2003; Mitani et al. 2010; Radford and Fawcett 2014). In this case, perhaps all group members would be expected to increase their grooming and sentinel behavior as our results indicate.

We found no discernible difference in the aftermath responses (sentinel activity, nearest-neighbor foraging distances) to rival playbacks and fecal presentations, contrary to our prediction that the former might indicate a more imminent threat and so elicit a stronger reaction. One possible explanation is that a playback does not fully replicate the circumstances relating to an approaching rival group. While rival playbacks did simulate an out-group threat, as there were relevant changes in behavior both during and after the manipulation, our playbacks were not followed by visual confirmation of a rival group. Without such visual validation shortly after hearing acoustic cues indicating a rival group presence, dwarf mongooses might not perceive the situation realistic of an imminent contest. Another potential reason for the lack of a difference between experimental treatment types is that encountering relatively fresh rival feces at a latrine might have generated similar anxiety to hearing another group. While acoustic cues might suggest an imminent encounter, uncertainty about the current location of the rival group that deposited the feces might cause equivalent anxiety and thus changes in vigilance and foraging decisions.

Conflict is recognized as a powerful selective force, yet relatively few studies have experimentally investigated the consequences of out-group conflict despite its prevalence in the animal kingdom. Both our field manipulations had the predicted effect of simulating an out-group threat—they resulted in clear changes in immediate and subsequent behavior—and therefore represent viable approaches for future work. Combining the ecological validity of studying animals in natural conditions with the power from controlled experimental testing, allows the generation of strong conclusions about the effects of out-group threats on within-group behaviors. Together, the 2 field manipulations provide an insight into 3 neglected avenues of research in this field: studying a broader range of postinteraction behaviors (beyond aggression and affiliation), looking at behavioral changes from an individual level and focusing on behavioral changes beyond the immediate aftermath (Radford et al. 2016). Future studies should adopt and expand on this approach, across a multitude of species and timeframes, to help unravel how out-group conflict shapes the lives of social species.

## Supplementary Material

arz095_suppl_Supplementary-Material
