# Supplementary material for: Experimental field evidence that out-group threats influence within-group behavior
Source: Behav Ecol. 2019 Jun 20;30(5):1425–35. doi: 10.1093/beheco/arz095 (PMC6765380; doi:10.1093/beheco/arz095)
Supplement: arz095_suppl_Supplementary-Material [file arz095_suppl_supplementary-material.docx]

**Experimental field evidence that out-group threats influence within-group behaviour**

**Amy Morris-Drake, Charlotte Christensen, Julie M. Kern & Andrew N. Radford**

**Supplementary Material**

**Supplementary Methods**

***Experimental location***

The level of threat from rival groups could vary depending on territorial location (Furrer et al. 2011; Brown 2013). To determine whether the location of experimental trials should to be taken into consideration when analysing the data, we first classified trials as either occurring in the core or periphery of each group’s territory. We calculated home ranges using the movement data collected from each group over a 5-month period prior to the experimental field season. The geographical waypoint data from the daily movement maps was transported into MapSource (software version 6.16.3) and then into QGIS (software version 2.14.4) for each group. The minimum convex polygon (MCP) algorithm from the plugin AniMove was used to estimate the full home range (using 100% of the data point fixes; as in Mattisson et al. (2013)) and the central ‘core’ of the home range (using the central 50% of data point fixes). The GPS points of experimental trials were added to each group’s territory to determine whether they fell within core (within MCP 50) or peripheral (outside MCP 50) regions as in Jędrzejewski et al. (2007).

To determine whether there was any unintentional bias towards running the experimental manipulations in the core or periphery of each group’s territory, Cochran Q and McNemar’s tests were carried out. Tests were conducted in RStudio 3.2.2 (R Development Core Team 2012), were two-tailed and considered significant at P < 0.05. First, a Cochran Q test was run for the three treatments in the Extra Experiment (see below); whether the trial was conducted in the core (Yes/No) was analysed. Second, two McNemar’s tests were run for Experiment 2 (see main manuscript) using the same response variable as above but split for those trials that involved playbacks and those that involved faecal presentations. We were unable to analyse the location data for Experiment 1 as only two out of seven groups differed in their sleeping burrow location between the two trials.

For both the Extra Experiment (see below) and Experiment 2, there was no unintentional bias towards running the experimental trials in the core or periphery of each group’s territory (Extra Experiment: Cochran Q test: Q = 2.8, df = 2, P = 0.2466; Experiment 2: McNemar test on playback trials: χ^2^  = 0.8, df = 1, P = 0.371; McNemar test on faecal presentation trials: χ^2^  = 0.25, df = 1, P = 0.617).

**Supplementary Discussion**

**Extra Experiment**

To determine whether there was a difference in response to playback of rival-group close calls and own-group close calls, each mongoose group (n=7) was exposed to the following three trials: playback of (i) herbivore (control) sounds, (ii) own-group close calls, and (iii) rival-group close calls. Recordings and playback track creation followed the same procedure outlined in the main text. Own-group tracks always consisted of calls no more than one month old.

The three trials to a given group were carried out on separate days, always in the morning, when the entire group was foraging in the same habitat type under calm conditions. Each trial was filmed with a HD Panasonic DMC-XS3EB-R video camera (Panasonic House, Berkshire, UK) and a GPS point was taken to mark the experimental location of each trial. Trial order was counterbalanced between groups and all three trials to a given focal group were completed within a two-week timeframe (mean ± SE = 4.4 ± 0.8 days, range = 3–9 days). Playbacks took place when there had been no natural alarm call or group disturbance (e.g. snake mob) for at least 10 min. Tracks were played from an Apple iPod (Apple, Cupertino, California, USA) through a portable SME-AFS field speaker (Saul Mineroff Electronics Inc., New York, USA), which was concealed in vegetation along their predicted foraging route. The mongooses were called to a location 5 m away from the loudspeaker using a small amount of hard-boiled egg and once 50% of the adults in the group were present the relevant playback track began. The following immediate responses were extracted from the video recordings of the trials: the number and identity of all the adult group members present at the start of the track; whether an individual looked and orientated (whole body pointing towards the loudspeaker) in the direction of the loudspeaker; and whether an individual directly approached the loudspeaker. Trials were not conducted if an intergroup interaction (IGI) occurred the morning of the trial and were abandoned if an alarm call, or any other group disturbance, occurred during the playback period (n=1).

To investigate whether there were sound-treatment differences in the proportion of individuals that looked and orientated towards the loudspeaker and the proportion of individuals that directly approached the loudspeaker, Friedman and Wilcoxen signed-ranks tests were carried out. Nonparametric tests were used, as the raw and transformed data did not conform with the assumptions of normality and homogeneity of variance. Tests were run using IBM SPSS Statistics for Windows, version 24 (IBM Corp, 2016), were two-tailed and considered significant at P < 0.05. The Monte Carlo method (based on 10,000 samples) was used to calculate significance due to unreliable p-value calculations when sample sizes are small.

The immediate responses to playback were significantly affected by sound treatment. Trial type had a significant effect on the proportion of individuals that looked and orientated towards the loudspeaker (Friedman test: χ^2^ = 7.75, df = 2, P = 0.021), and the proportion of individuals that directly approached the loudspeaker (χ^2^ = 8.24, df = 2, P = 0.016). For both response variables, there was no significant difference between the two non-threat trials (control and own-group playback): proportion looked and orientated (Wilcoxon matched-pairs signed ranks test: Z = 1.473, N = 7, Monte Carlo P = 0.248); and proportion that directly approached (Z = 1.089, N = 7, Monte Carlo P = 0.371). However, there was a significantly stronger response to rival-group playback compared to the two non-threat trials (control and own-group playback): a greater proportion looked and orientated during rival-group playback compared to own-group (Z = 2.201, N = 7, Monte Carlo P = 0.031) and control playback (Z = 2.201, N = 7, Monte Carlo P = 0.030); and a greater proportion directly approached during rival-group playback compared to own-group (Z = 2.032, N = 7, Monte Carlo P = 0.045) and control playback (Z = 2.207, N = 7, Monte Carlo P = 0.031).

**References**

Brown M. 2013. Food and range defence in group-living primates. Anim Behav*.* 85:807

–816.

Furrer RD, Kyabulima S, Willems EP, Cant MA, Manser MB. 2011. Location and group size influence decisions in simulated intergroup encounters in banded mongooses. Behav Ecol. 22:493–500.

Jędrzejewski W, Schmidt K, Theuerkauf J, Jędrzejewska B, Kowalczyk R. 2007. Territory size of wolves Canis lupus: linking local (Białowieża Primeval Forest, Poland) and Holarctic-scale patterns. Ecography. 30:66–76.

Mattisson J, Sand H, Wabakken P, et al. 2013. Home range size variation in a recovering wolf

population: evaluating the effect of environmental, demorgraphic, and social factors.

Oecologia. 173:813–825.
